# Supplementary material for: Assessment of the Correlation and Diagnostic Accuracy between Cerebrospinal Fluid and Plasma Alzheimer’s Disease Biomarkers: A Comparison of the Lumipulse and Simoa Platforms
Source: Int J Mol Sci. 2024 Apr 23;25(9):4594. doi: 10.3390/ijms25094594 (PMC11083365; doi:10.3390/ijms25094594)
Supplement: Supplementary file 1 [file ijms-25-04594-s001.zip › ijms-2925620-supplementary.pdf]

SUPPLEMENTARY DATA

Assessment of the Correlation and Diagnostic Accuracy between CSF and Plasma AD Biomarkers: A Comparison of the Lumipulse and Simoa Platforms

Farida Dakterzada[1^], Raffaela Cipriani[2^], Ricard López-Ortega[3], Alfonso Arias[1], Iolanda Riba-Llena[1], Maria Ruiz-Julián[1], Raquel Huerto[1], Nuria Tahan[1], Carlos Matute[2,4,5], Estibaliz Capetillo-Zarate[2,5,6,7^^], Gerard Piñol-Ripoll[1,8^^*]

(1) Cognitive Disorders Unit, Cognition and Behaviour Study Group, Santa Maria University Hospital, IRBLleida, Lleida, 25198, Spain

(2) Achucarro Basque Center for Neuroscience, Leioa, 48940, Spain

(3) Laboratori Clínic ICS, Hospital Universitari Arnau de Vilanova, Lleida, 25198, Spain

(4) Department of Neurosciences, Faculty of Medicine and Nursery, University of the Basque Country (UPV/EHU), Leioa, 48940, Spain

(5) CIBERNED, Centro de Investigación Biomédica en Red Enfermedades Neurodegenerativas, Madrid, Spain

(6) Department of Neurosciences, Faculty of Pharmacy, University of the Basque Country (UPV/EHU), Vitoria-Gasteiz, 01008, Spain

(7) IKERBASQUE, Basque Foundation for Science, Bilbao, 48009, Spain

(8) Departament de Medicina Experimental, Facultat de Medicina, Universitat de Lleida (UDL), Spain

Supplementary Table S1. Correlation coefficient between CSF AD biomarkers measured by Lumipulse and the same markers quantified in plasma with the Simoa or Lumipulse platforms

		Correlation coefficient	*p*-value
Between Lumipulse CSF and Simoa plasma	Aβ42	0.107	0.238
	Aβ40	0.023	0.801
	Ptau181	0.363	< 0.001
	Ttau	-0.024	0.79
	Aβ42/40	0.211	0.019
	Ptau181/Aβ42	0.346	< 0.001
	Ttau/Aβ42	0.046	0.61
	Simoa Ptau181 & Lumipulse CSF Aβ42	-0.269	0.002
	Simoa Ptau181 & Lumipulse CSF Aβ42/40	-0.4	< 0.001
Between Lumipulse CSF and Lumipulse Plasma	Aβ42	0.251	0.005
	Aβ40	0.072	0.431
	Ptau181	0.33	< 0.001
	Aβ42/40	0.423	< 0.001
	Ptau181/Aβ42	0.332	< 0.001
	Lumipulse plasma Ptau181 & Lumipulse CSF Aβ42	-0.226	0.011
	Lumipulse plasma Ptau181 & Lumipulse CSF Aβ42/40	0.35	< 0.001
